# Characteristics of Nontyphoid *Salmonella* Isolated from Human, Environmental, Animal, and Food Samples in Burkina Faso: A Systematic Review and Meta-Analysis

**DOI:** 10.3390/antibiotics13060556

**Published:** 2024-06-13

**Authors:** Kuan Abdoulaye Traore, Abdoul Rachid Aboubacar-Paraiso, Soutongnooma Caroline Bouda, Jean Bienvenue Ouoba, Assèta Kagambèga, Pierre Roques, Nicolas Barro

**Affiliations:** 1 Laboratoire de Biologie Moléculaire, d’Epidémiologie et de Surveillance des Bactéries et Virus Transmissibles par les Aliments (LaBESTA), Université Joseph KI-ZERBO (UJKZ), Ouagadougou 03 BP 7021, Burkina Faso; rachidparaiso56@gmail.com (A.R.A.-P.);; 2 Laboratoire Sciences de la Vie et de la Terre (LaSVT), Université Norbert ZONGO (UNZ), Koudougou BP 376, Burkina Faso; 3 Centre Universitaire de Manga (CUM), Université Norbert ZONGO (UNZ), Koudougou BP 376, Burkina Faso; 4 Department of Biology, Institute of Sciences (IDS), Ouagadougou 1757, Burkina Faso; 5 Virology Unit, Institut Pasteur de Guinée (IPGui), Conakry 4416, Guinea; pierre.roques@pasteur.fr

**Keywords:** *Salmonella* spp., nontyphoidal, serotypes, resistance phenotype, systematic review, meta-analysis, Burkina Faso

## Abstract

*Salmonella* is one of the world’s leading causes of zoonotic and foodborne illnesses. Recently, antimicrobial resistance (AMR) has become one of the most critical challenges to public health and food safety. Herein, we employed a meta-analysis to determine the pooled prevalence and spatiotemporal distribution of serovars and antimicrobial resistance in NTS in Burkina Faso. To find eligible articles, a comprehensive literature search of PubMed, African Journals Online, ScienceDirect, Google Scholar, and the gray literature (university libraries) in Burkina was conducted for the period from 2008 to 2020. Studies meeting the inclusion criteria were selected and assessed for risk of bias. To assess the temporal and spatial relationships between serotypes and resistant strains from humans, animals, food, and the environment, a random-effects statistical model meta-analysis was carried out using the Comprehensive Meta-Analysis Version 3.0 program. The NTS prevalence rates were 4.6% (95% CI: 3–7) and 20.1% (95% CI: 6.6–47.4) in humans and animals, respectively, and 16.8% (95% CI: 10.5–25.8) and 15.6% (95% CI: 8.2–27.5) in food and the environment, respectively. Most NTS serovars were *S.* Derby, reported both in food and animals, and *S.* Typhimurium, reported in humans, while *S.* Croft II, *S.* Jodpur II, and *S.* Kentucky were the most prevalent in the environment. NTS isolates were highly resistant to erythromycin, amoxicillin, cefixime, and cephalothin, with a pooled prevalence of multidrug resistance of 29% (95% CI: 14.5–49.5). The results of this review show a high diversity of *Salmonella* serotypes, as well as high antibiotic resistance in *Salmonella* isolates from animal, human, food, and environmental samples in Burkina, calling for a consolidated “One Health” approach to better understand the drivers of pathogen emergence, spread, and antimicrobial resistance, as well as the formulation of intervention measures needed to limit the risk associated with the disease.

## 1. Introduction

Urbanization and the impact of socioeconomic development have become the main determinants of the health of the world’s population and the hygiene of the environment in which they live [1]. A lack of hygiene thus provides bioecological conditions favorable for the development of pathogenic germs responsible for numerous diseases, particularly gastroenteritis, the main manifestation of which is diarrhea [2]. There are multiple causes of gastroenteritis, including bacteria, viruses, and parasites [3]. Among bacterial etiological agents, *Salmonella* spp. is among the most problematic food-borne and zoonotic pathogens threatening general health and well-being [4].

*Salmonella* is the main cause of acute gastroenteritis in many countries, and salmonellosis remains a major public health problem worldwide, particularly in developing countries [5]. It manifests primarily as mild diarrhea, also known as food poisoning [6].

The global burden of nontyphoidal *Salmonella* gastroenteritis is estimated to be 93.8 million cases of gastroenteritis each year, with 155,000 deaths [7]. Moreover, it remains the second most reported agent of gastrointestinal infections in humans in Europe and North America [8].

In Africa, it has consistently been reported as a leading cause of bacteremia in immunocompromised individuals, infants, and newborns [9]. Nontyphoidal *Salmonella* serotypes (NTS) are among the most frequent causes of bloodstream infections in sub-Saharan Africa, with approximately 680,000 deaths per year, mainly in children under five years of age [10]. Although more than 2700 serotypes of *Salmonella enterica* have been identified [11], the serotypes most commonly implicated in invasive disease are *S.* Typhimurium, *S.* Enteritidis, and *S.* Dublin [12]. Nevertheless, in recent years, data have revealed the emergence of infection due to the consumption of raw fruits and vegetables associated with rare serotypes from the environment [13].

Prevention of salmonellosis (caused by nontyphoidal *Salmonella*) is difficult due to its complex epidemiology and multiple modes of transmission [14]. Human disease can result from exposure to numerous sources, such as infected animals, contaminated foodstuffs, contaminated water, and direct contact with an infected environment or direct contact between humans [15].

Poultry and pork meats, eggs, dairy products, and green vegetables contaminated with manure or irrigation water are the agents or risk factors most cited in the transmission of this bacterium [9].

The public health problem associated with nontyphoidal *Salmonella* is antimicrobial resistance. The emergence of multiple antibiotic-resistant *Salmonella* strains adds another important dimension to the challenge of controlling *Salmonella*, as resistant variants can compromise the ability to treat human infections, a particularly important issue in the case of systemic infections [14]. Several clones of multidrug-resistant *Salmonella* emerged in the late 1990s and early 2000s, and since then, their prevalence in humans, domestic animals, and other wildlife has spread globally [16,17,18]. Recently, the increasing prevalence of *Salmonella* multi-resistant to clinically important antimicrobials, such as fluoroquinolones and third-generation cephalosporins, has become an emerging problem worldwide [19,20,21,22].

Despite the knowledge of the prevalence of *Salmonella* and its antimicrobial resistance profile, which is mostly reported by individual and local surveillance study(s), comprehensive and robust studies of the prevalence and antimicrobial resistance pattern in Burkina are poorly characterized [23,24,25,26]. Therefore, concise information from a systematic review is more helpful for scientific users to identify gaps for additional studies and for policymakers to develop prevention and control strategies based on the scientific information provided. Thus, this meta-analysis includes a comprehensive evaluation of the scientific literature published between 2008 to 2022 on the state of knowledge on the prevalence, serovars, and antimicrobial resistance phenotypes of NTS strains from studies carried out in Burkina Faso.

## 2. Results

### 2.1. Overview of the Selected Studies

**Figure 1:**
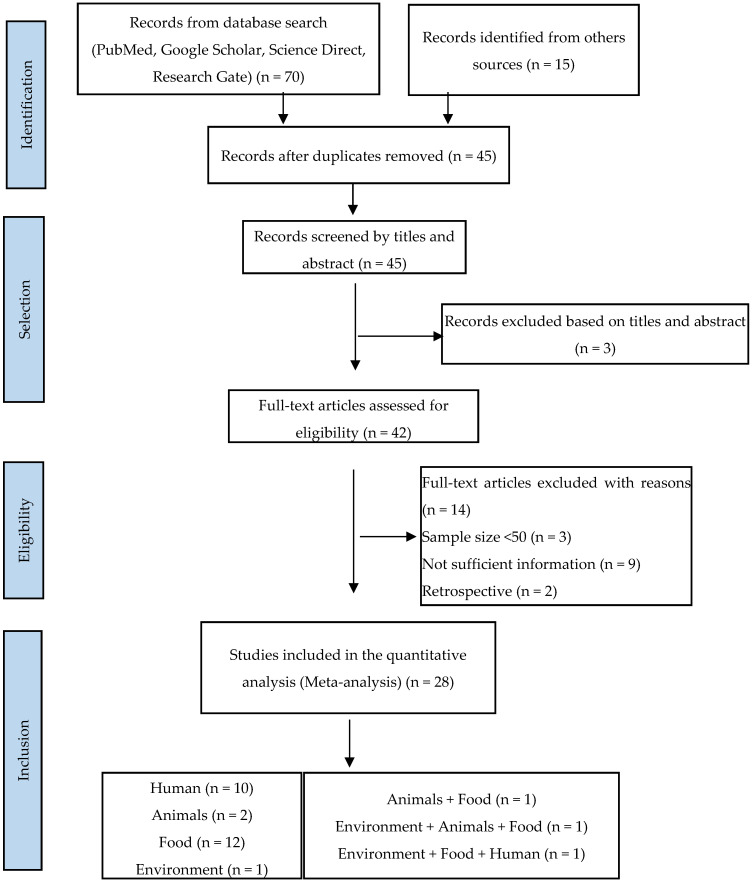
PRISMA flowchart illustrating the process of identifying, screening, and selecting the eligible articles used in this study.

A total of 85 articles were found in our initial research, of which 70 publications were from Google Scholar, PubMed, Science Direct, and Research Gate, and 15 were from other sources. Of the total, 28 publications (33%) considered suitable for inclusion in this review were identified, including 23 eligible studies from a database search (85.71%) and 5 from the literature searches at university libraries (17.86%). A flow diagram of the literature search and selection of eligible studies is presented in Figure 1.

### 2.2. Study Characteristics of Eligible Studies

The following are the characteristics of the studies that are eligible: articles published primarily on the quantitative prevalence of *Salmonella* serovars/isolates in humans, animals (chickens, ducks, cows, pigs, sheep, etc.), foods (lettuce, sandwiches, beef, mutton, poultry, etc.), and the environment in Burkina Faso; the type of samples and method of diagnostics used; the exact number of samples, as well as the number of positives tested; articles reported in English only; and antibiotic resistance. All of the journal articles were published between 2008 and 2022 (Table 1).

**Figure 2:**
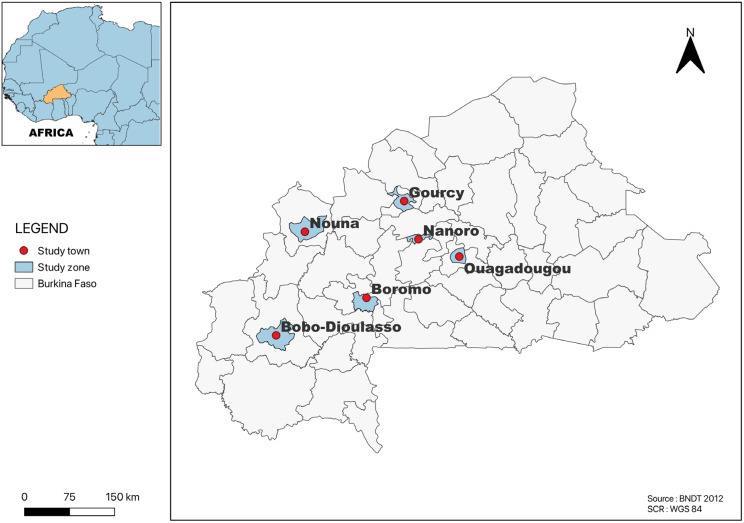
Map of Burkina Faso showing the study area.

All of the studies included in this review were carried out in towns located in four regions on the country, Centre, Nord, Hauts-Bassins and Boucle du Mouhoun. Several studies were from Ouagadougou (*n* = 24), followed by Area 1 (Gourcy and Boromo (*n* = 3)); Area 2 (Bobo-Dioulasso and Ouagadougou (*n* = 2)); Area 3 (Ouagadougou, Gourcy and Boromo (*n* = 1)); Nanoro (*n* = 1); and Nouna in the province of Kossi (*n* = 1) (Figure 2). The most common method for determining the AR profiles of *Salmonella* serotypes isolated from all of the studies included in this systematic and meta-analysis study was disk diffusion.

**Figure 3:**
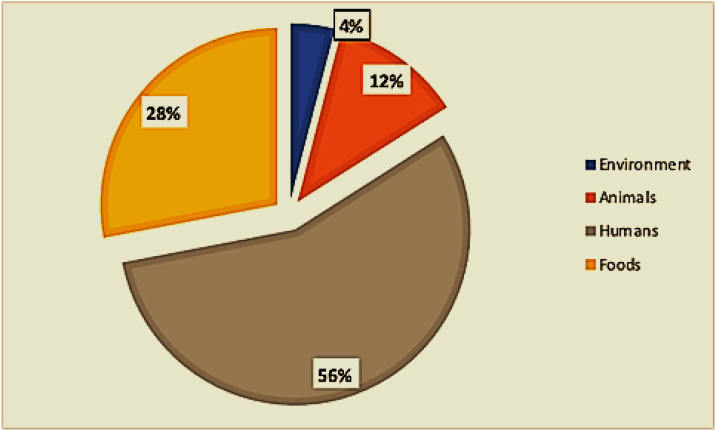
Distribution of sample types.

Of the twenty-eight included studies, twelve (12) were from food sources, eleven (11) were from clinical sources (mainly children under 5 years of age), two (02) were from animal sources, one (01) was from environmental sources, and one (01) included both animal and environmental sources (Figure 3). One study examined food, human, and environmental hosts, and one study included hosts of both food and animal origin (Figure 1).

### 2.3. Prevalence Based on Sources and Locality (Sampling Sites)

A high degree of heterogeneity was observed in studies carried out on different sources (Table 2). For the animals, pooled heterogeneity was observed [20.1% (95% CI: 6.6–47.4), I^2^ = 98.48]; for food [16.6% (95% CI: 9.9–26.4), I^2^ = 96.10]; for humans [5.4% (95% CI: 4.8–6), I^2^ = 91.94]; and for the environment [15.6% (8.2–27.5), I^2^ = 83.20]. Egger’s test revealed no statistically significant publication bias in the estimation of the prevalence of *Salmonella* from three sources: humans (*p* = 0.17), food (*p* = 0.14), and the environment (*p* = 0.45). However, one source of heterogeneity was identified as significant (*p* = 0.03). The pooled prevalence of *Salmonella* isolates found in Ouagadougou was 23.1% (95% CI: 21.9–24.4, I^2^ = 98.25], followed by area 2 [18.4% (95% CI: 14.6–22.8), I^2^ = 0.00] and area 1 [6.4% (95% CI: 4.9–8.2), I^2^ = 70.92]. However, some study areas and cities (areas 3, Nanoro, and Nouna) were not included in the meta-analyses due to the low number of studies.

### 2.4. Pooled Prevalence and Distribution of *Salmonella* Serotypes

In this study, A total of 145 serotypes out of 919 isolates were identified in humans, food, animals, and the environment (Supplementary Material S2). Table 3 shows the pooled prevalence and distribution of *Salmonella* serotypes. The prevalence of *S.* Derby was 23.92% (95% CI: 20.76–27.39), followed by *S.* Croft II at 18.75% (95% CI: 6.17–44.75), *S.* Jodhpur II at 18.75% (95% CI: 6.17–44.75), *S.* Tennessee at 16.35% (95% CI: 10.42–24.73), *S.* Muenster at 10.54% (95% CI: 7.99–13.80), *S.* Typhimurium at 10.42% (95% CI: 7.78–13.83), *S.* Tilene at 10.03% (95% CI: 4.94–19.28), *S.* Jodhpur at 9.77% (95% CI: 3.71–23.34), *S. Chester* at 9.07% (95% CI: 7.01–11.65), and *S.* Hato at 8.31% (95% CI: 6.38–10.75 The prevalence of the remaining *Salmonella* species was less than 8%. The prevalence of *Salmonella* varies considerably between different sources (Table 3). In humans, *S.* Typhimurium was observed at 21.27%, followed by *S.* Poona (15.83%). *S.* Derby was highly prevalent in animals (14.62%), followed by *S.* Muenster (11.26%). Considering environmental sources, *S.* Croft II, *S.* Jodhpur II, and *S.* Kentucky accounted for 18.75%, followed by *S.* Poona (16.13%). S. Derby was highly prevalent in food (30.45%), followed by *S.* Tennessee (27.74%). This study investigated the antimicrobial resistance of different *Salmonella* serotypes (Table 4). Serotype Typhimurium is the only multidrug resistance (MDR) strain found in humans, animals, and food. Moreover, MDR was revealed for the serotypes Brancaster, Enteritidis, Kentucky, Chester, and Derby in food; Hato and Urbana in animals; and Virchow, Poona, Duisburg, and Ouakam in humans.

### 2.5. Antibiotic Resistance Profile of *Salmonella* spp. Isolates

**Figure 4:**
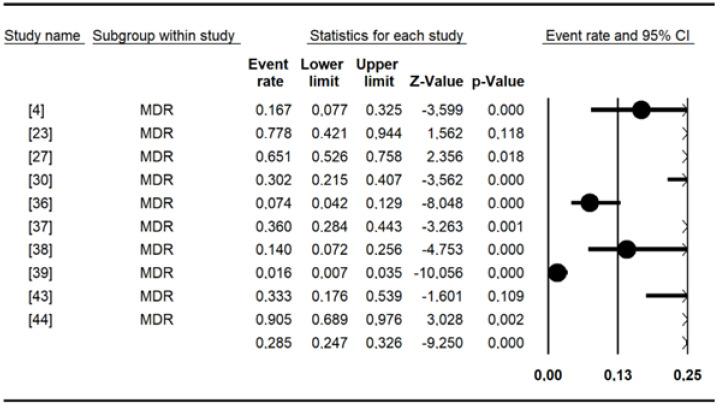
Forest plot showing the pooled estimates of multidrug resistance in studies conducted in Burkina Faso.

The pooled prevalence of antibiotic resistance for *Salmonella* included in this meta-analysis (Table 5) was as follows: erythromycin had a high prevalence of (98.3%), amoxicillin (68.69%), cefixime (56.24%), cephalothin (54.65%), amoxicillin and clavulanic acid (42.06%), cefepime (38.99%), tetracycline (36.50%), tobramycin (33.68%), cefotaxime (29.44%), colistin sulfate (28.49%), piperacillin (27.49%), and ampicillin (20.84%). The remaining data are shown in Table 5. Chloramphenicol, nalidixic acid, and tetracycline were the most commonly tested antibiotics, with more than 10 studies each. Overall, the MDR recorded for *Salmonella* spp. was 29% [95% CI: 15–50] (Figure 4).

### 2.6. Publication Bias

**Figure 5:**
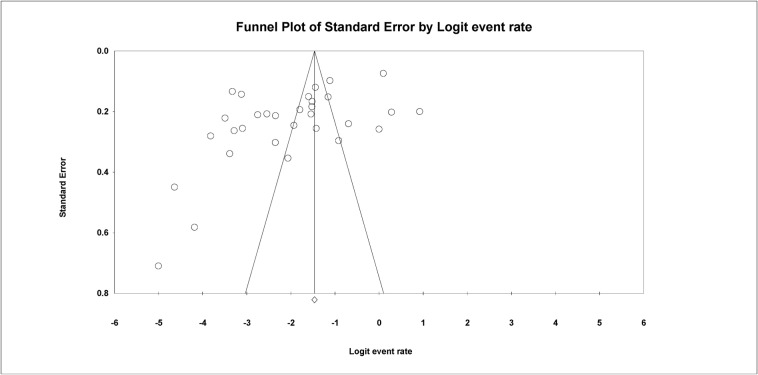
Funnel plot with 95% confidence limits of the pooled prevalence of the studies conducted in Burkina.

The Begg and Mazumdar rank correlation test demonstrated no significant publishing bias for all parameters (*p* value > 0.05 (Table 2 and Figure 5).

## 3. Discussion

*Salmonella* is globally one of the leading causes of human death among infectious diarrheal diseases, and antimicrobial-resistant strains of *Salmonella* are a worldwide health concern [49,50,51]. Understanding the epidemiological status of *Salmonella* is thus crucial for controlling this foodborne pathogen [52]. In Burkina Faso, primary research on the prevalence of *Salmonella* in humans, animals, food, and the environment, as well as its antimicrobial resistance status, have been reported. This meta-analysis focused on comprehensive and robust assessments.

This study examined 28 published articles from Burkina Faso, and the majority of the studies included were published between 2008 and 2022. This study revealed that the highest pooled prevalence of *Salmonella* was 23% (95% CI: 21.8–24.4) in Ouagadougou, followed by area 2 [18.4% (95% CI: 14.6–22.8)] and area 1 [6.4% (95% CI: 4.9–8.2)]. The number of studies included might be a possible reason for the region wise pooled prevalence variation. For instance, only a single study each was found in area 3, Nanoro and Nouna, which may not reflect the true pooled prevalence.

The pooled prevalence in the present systemic review and meta-analysis was 4.6% in humans, 20.1% in animals, 16.8% in food, and 15.6% in the environment. One possible reason for the higher prevalence of *Salmonella* in animals and food may be associated with unhygienic husbandry practices [53,54]. Moreover, meat handling practices in slaughterhouses and butcheries are generally unhygienic. The survival of *Salmonella* in both soil and water may be a potential cause of its high prevalence in the environment [55].

In the present study, the pooled prevalence of *Salmonella* among human samples was similar to that reported in studies in West Africa [56] and Ethiopia [57], which were 5.21% (95% CI: 3.37–7.06) and 4.8% (95% CI: 3.9, 5.9), respectively.

However, higher pooled prevalence reports were reported in Morocco (17.9%, 95% CI: 5.7–34.8%), Tunisia (10.2%, 95% CI: 4.3–18.0%), and Sudan (9.2%, 95% CI: 6.5–12.2%). [58]. This variation might be due to the type of population studied, the study period, the isolation method, and seasonal and geographical variations, but it may also increase community awareness of personal and environmental hygiene [59,60].

According to the subgroup analysis, the pooled prevalence of *Salmonella* in animals was 20.1%, which is lower than that reported in Chikwawa and Malawi in pigs (24.6%) [61], but higher than that observed in Nigeria (8.3%) [62], Australia (4%), and Japan (0.5%) [63,64]. The levels of *Salmonella* contamination in animals can vary depending on the country, the nature of the production system and the specific control measures in place [39]. In Burkina Faso, the majority of these animals are still raised under nonmodern conditions. Animals are grazed in an environment with feed of poor microbiological quality. This is more common in poultry farming. These practices undoubtedly lead to *Salmonella* contamination in animals [65].

The pooled prevalence estimate of *Salmonella* in food was similar to, but slightly greater than, that reported for Mali (12.80%) in poultry carcasses [66]. Other studies have reported prevalence rates of 17.7% in Pakistan and 14.4% in Iraq [67]. These results point to food contamination due to noncompliance with good hygiene practices [68]. *Salmonella*, which is usually present in the intestinal tract of animals, can contaminate food during processing procedures and can survive after a cooking process where the cooking time is too short or the temperature is not high enough [69,70]. A study conducted in Burkina Faso showed that 31 of the raw ingredients used to prepare sandwiches were contaminated with *Salmonella* from the water used to wash the lettuce [38]. This contamination can be of environmental, animal, or human origin during the cultivation, harvesting, or handling of plants prior to consumption [38,71].

The environmental samples identified in our meta-analysis consisted mainly of water (well water, canals, taps, fountains, and reservoirs). The reported clustered prevalence of *Salmonella* corroborates that which was reported in Senegal in irrigation water, which was 17% [72]. Increasing evidence indicates that irrigation water is a source (or a vehicle) for the transmission of *Salmonella* [73,74,75,76]. This result is worrying, given that in Ouagadougou, all kinds of wastewater (urban or industrial wastewater) are used in an unplanned and uncontrolled way in urban and peri-urban agricultural activities, including the irrigation of vegetables consumed raw (lettuce in particular) [32]. Moreover, the lack of mineral matter in the soil and the impossibility for some producers to obtain chemical fertilizers have led them to use animal manure as fertilizer, which is more economical and beneficial for the environment. However, several studies have shown that animal excrement is a major vector for bacterial contamination of garden products [77].

The measurement of trends in serovars over time can provide information about emerging serotypes and about the efficacy of prevention [78].

In this study, the majority of strains were isolated from animals (49.8%, 458/919) and food (38.41%, 353/919). This finding is in agreement with a previous report in South Africa [79]. In the present study, NTS from both humans, the environment, food, and animals dominated by 7.69% in *S.* Kentucky, followed by 7.57% in *S.* Cubana, 6.67% in *S.* Poona, and 4.34% in *S.* Senftenberg. *Salmonella* has a broad host range, and hence is considered a universal pathogen. Each serovar has a different ability to adapt to the host environment and cause virulency. Some *Salmonella* serovars are restricted to one host, whereas some have a broad host spectrum [80]. For instance, *S.* derby was isolated from animals and food in 23.92% of the patients. *S.* Croft II and *S.* Jodhpur II were observed only in the environmental samples. In this review, we found that *S.* Typhi was highly prevalent in humans and was also reported in Ethiopia [81], China [82], the Middle East, and North Africa [58]. According to reports from sub-Saharan African nations, *S.* Typhimurium is one of the invasive forms of NTS, especially among susceptible people, such as those with HIV, malnourished children, and malaria [10,83]. Furthermore, MDR strains mainly existed in *S.* Typhimurium. This serovar was also detected in both animal and food samples, even though it was not highly prevalent. Notably, no environmental cases of *S.* Typhi were found.

The main serotypes found in both animal and food samples in this study included *S.* Derby, *S.* Hato, and *S.* Chester. Poona was the most common serotype detected in both human and environmental samples. *Salmonella*, regardless of serovar, can persist in dry environments as well as in water for many weeks to months. Moreover, environmental factors may influence the survival of serovars and could contribute to within- and between-host species differences [84]. Therefore, the ability of a pathogen to spread disease in populations is influenced by host adaptation in many ways [52,85].

Our meta-analysis revealed high NTS resistance against erythromycin (98.3%), amoxicillin (68.69%), cefixime (56.24%), cephalothin (54.65%), and amoxicillin and clavulanic acid (42.06%) for isolates from all source-specific samples. These levels of resistance may be due to the indiscriminate application of antibiotics in human and animal health and food production, followed by the leaching of antibiotics into the environment [86,87]. High resistance of *Salmonella* to erythromycin was observed by Ramatla et al. in a systematic review in South Africa [88]. This is probably due to its veterinary use as a feed supplement and/or treatment, particularly in the poultry sector [89,90]. Amoxicillin is one of the most commonly used antibiotics worldwide for treating salmonellosis [79].

High resistance to this antibiotic was found in a study carried out in the Middle East, where the rate of resistance to amoxicillin was 100% [91].

However, with the emergence of resistant isolates, traditional antibiotics have been replaced by cephalosporins [92]. In the present study, resistance to cefixime and cephalothin was 56.24% and 54.65%, respectively. A meta-analysis carried out in Saudi Arabia reported a high prevalence of 90.9% for cephalothin [93] and 9% for cefixime in Iran [94]. This difference could be explained by the level of antibiotic use, which varies from one country to another.

Ten studies reported multidrug resistance to antimicrobials in the present study. Our results (29%) are in line with those of a previous study [88] in which multidrug resistance (MDR) (28.5%) was detected. It has been documented that infections caused by multidrug-resistant strains are more severe than those caused by susceptible strains [95]. Thus, MDR strains present in food (Typhimurium, Brancaster, Enteritidis, Kentucky, Chester, and Derby) and animals (Typhimurium, Hato, Urbana) could directly threaten human health, as they can cause diseases that are difficult to cure. In addition, integrons, which are mobile genetic elements often associated with multiresistant *Salmonella* phenotypes, are thought to play a key role in the spread of antimicrobial resistance genes among Gram-negative bacteria [96]. The present study shows the urgent need to control the use of antibiotics in veterinary and human medicine to limit the spread of multidrug-resistant *Salmonella* strains.

This study has the following limitations. The number of studies from some localities was very high compared with that from others, which may have influenced the overall estimate. In addition, few studies on this topic in the environment are available. Only one study was included, which limited comparisons of the prevalence and resistance profiles. Some studies reported the total number of isolates without showing the number of individual serotypes. Resistance genotypes were not included in this meta-analysis due to the small number of studies carried out.

## 4. Materials and Methods

### 4.1. Study Design

The current systematic review and meta-analysis were performed according to the Preferred Reporting Items for Systematic Reviews and Meta-Analyses (PRISMA) guidelines for selection criteria, literature search, statistical analysis, and data extraction [97].

### 4.2. Search Strategy

The literature search was performed on four databases, namely, PubMed, Google Scholar, Science Direct, and African Journals Online, and manually by obtaining hard copies of locally published articles directly from authors and local libraries.

The following keywords were used to search for the articles: antibiotic resistance AND antibiotic AND drug resistance AND bacterial resistance AND multidrug resistance AND *Salmonella* species AND human OR animal AND environment OR food AND nontyphoid *Salmonella* AND Burkina Faso. We conducted our last search on November 2022.

### 4.3. Inclusion and Exclusion Criteria

All of the eligible studies included met the following inclusion criteria in the review: articles published primarily on the prevalence of nontyphoidal *Salmonella* spp. in the environment, foods, animals, and humans in Burkina Faso; the type of samples and method of diagnostics used (approved for the detection and antibiogram of *Salmonella* spp.); the exact number of samples as well as the number of positives tested; and articles reported in English only on serotypes and antimicrobial resistance published between 2008 and August 2022. Studies were excluded if they were not performed in Burkina Faso. In addition, reviews with smaller sample sizes (≤50) and articles not reported in English were excluded.

### 4.4. Study Selection

Records identified from various sources with the search strategies were exported to Endnote reference manager software version 8. Duplicate records were identified, recorded, and removed. The titles and abstracts of journal articles were examined and downloaded. Two authors independently screened the title and abstracts with the predefined inclusion criteria. The full versions of potentially relevant articles were obtained to evaluate their eligibility for final inclusion. During the screening process, an additional author was on standby to resolve any discrepancies that may arise with study selection.

### 4.5. Data Extraction and Data Collection

Data including names of authors, publication year, location, total sample size, and standard methods to detect the antibiotic resistant, number of positive samples were collected from each publication independently. Then, they were entered into a spreadsheet (Microsoft Excel^®^), tables, and a word document template. Only *Salmonella* species/isolates/serotypes specific journal articles were included in the meta-analysis. The studies with insufficient data were excluded. In addition, review articles and studies with an abstract, but without a full text, were excluded. The papers that included the drug susceptibility test, and number of multidrug resistance strains were considered and included in this study. 

### 4.6. Data Analysis and Assessment of Risk of Bias

For each study, the prevalence, effect size, and 95% CIs were calculated as point estimates. A comprehensive meta-analysis was used for all of the statistical analyses, version 3.0 (CMA) program (https://www.meta-analysis.com/, accessed on 19 November 2022). The software was used to generate the pooled estimates, Cochran’s Q, *p* values, and forest plots. I^2^ (level of inconsistency) was used to assess the heterogeneity of the studies (Cochran’s Q). I^2^ values of 25–50%, 51–75%, and >75% were considered to represent low, medium, and high heterogeneity, respectively. Low I^2^ values suggest that variability between estimates is consistent with random variation [13]. Interstudy heterogeneity was considered significant if the *p* value of Cochran’s Q test was less than 0.05. The Begg and Mazumdar test was used to investigate the possibility of propagation bias. Funnel plots were used to assess publication bias [98].

## 5. Conclusions

This systematic review and meta-analysis report the trends and distributions of *Salmonella* serotypes and antibiotic resistance in humans, animals, and food in Burkina Faso. It also highlights the lack of published scientific data on antibiotic resistance of *Salmonella* strains in the environment. 

The pooled prevalence of NTS was greatest for animals and food. The predominant NTS serotypes were *S.* Cubana, *S.* Typhimurium, *S.* Derby, *S.* Hato, *S.* Chester, and *S.* Poona. This predominance of emerging isolates is a real public health problem. NTS isolates also were highly resistant to erythromycin, amoxicillin, cefixime, and cephalothin, with a fairly high rate of multidrug resistance. In view of this situation, a consolidated “One Health” approach to the human, animal, food, and environmental sectors is needed to establish a safety net against salmonellosis in Burkina Faso.

## Figures and Tables

**Table 1:** Descriptive characteristics of the included studies.

Author, Year	Study Period	Locality	Samples Type	Lab Test Used	Antibiogram	Sample Size	Diagnosis Cases
[4]	2017–2018	Ouagadougou	Food	Culture	DDM	201	36
[27]	2019	Bobo-Dioulasso, Ouagadougou	Food	Culture	DDM	245	44
[28]	2008–2009	Ouagadougou	Food	Culture	DDM	150	19
[26]	2009–2015	Ouagadougou, Gourcy, Boromo	Food	Culture	DDM	78	26
[29]	2010	Ouagadougou	Food	Culture	DDM	100	57
[30]	2011–2012	Ouagadoudou	Food	Culture	DDM	450	86
[31]	2011–2012	Ouagadougou	Food	Culture	DDM	300	2
[32]	2021	Ouagadougou	Food	Culture	DDM	80	9
[33]	2017	Ouagadougou	Food	Culture	Nd	123	88
[34]	2011–2012	Ouagadougou	Food	Culture	DDM	200	3
[35]	2013–2014	Ouagadougou	Food	Culture	Nd	60	30
[15]	2020	Ouagadougou	Food	Culture	Nd	159	28
[36]	2018–2020	Ouagadougou	Food	Culture	DDM	1052	148
[37]	2016	Ouagadougou	Animals	Culture	DDM	563	139
[38]	2008–2009	Ouagadougou	Animals	Culture	DDM	238	57
[27]	2019	Bobo-Dioulasso, Ouagadougou	Animals	Culture	DDM	98	19
[39]	2010	Ouagadougou	Animals	Culture	DDM	729	382
[40]	2010	Ouagadougou	Animals	Culture	DDM	370	16
[38]	2008–2009	Ouagadougou	Environment	Culture	DDM	218	31
[26]	2009–2015	Ouagadougou, Gourcy, Boromo	Environment	Culture	DDM	56	16
[41]	2008–2009	Ouagadougou	Environment	Culture	DDM	138	12
[42]	2009–2010	Ouagadougou	Humans	Culture	DDM	343	25
[23]	2009–2010	Gourcy, Boromo	Humans	Culture	Nd	275	9
[26]	2009–2015	Ouagadougou, Gourcy, Boromo	Humans	Culture	DDM	416	15
[43]	2009–2010	Gourcy, Boromo	Humans	Culture	DDM	400	24
[44]	2012–2013	Ouagadougou	Humans	Culture	DDM	711	21
[45]	2015	Nanoro	Humans	Culture	DDM	1207	51
[46]	2013	Ouagadougou	Humans	Culture	Nd	200	3
[47]	2015	Ouagadougou	Humans	Culture	Nd	1674	58
[25]	2013–2017	Province de Kossi	Humans	Culture + PCR	Nd	605	13
[48]	2010	Gourcy, Boromo	Humans	Culture	Nd	275	24
[24]	2013–2015	Ouagadougou	Humans	Culture	Nd	315	53

Legend: DDM = Kirby Bauer Disc Diffusion Method, Nd = Not performed, PCR = Polymerase Chain Reaction.

**Table 2:** Pooled prevalence of *Salmonella* spp. from the environment, animals, food, and humans and sampling sites.

Risk Factors	Pooled Estimates		Measure of Heterogeneity			Publication Bias Begg and Mazumdar Rank *p* Value
	Number of Studies	Pooled Prevalence (95% CI)	Q	I^2^	*p* Value	
Sources						
Humans	11	5.4 (4.8–6)	124.138	91.94	0.00	0.64
Animals	04	20.1 (6.6–47.4)	196.905	98.48	0.00	0.30
Food	14	16.6 (9.9–26.4)	333.514	96.10	0.00	0.10
Environment	03	15.6 (8.2–27.5)	11.907	83.20	0.003	1.00
Locality						
Ouagadougou	24	23.1 (21.9–24.4)	1152.833	98.00	0.00	0.06
Area 1	03	6.4 (4.9–8.2)	6.877	70.92	0.032	1.00
Area 2	02	18.4 (14.6–22.8)	0.095	0.00	0.76	-
Area 3	01	10.4 (8.1–13.2)	-	-	-	-
Nanoro	01	4.2 (3.2–5.5)	-	-	-	-
Nouna	01	2.1 (1.3–3.7)	-	-	-	-

Legend: Area 1: Boromo and Gourcy; Area 2: Bobo-Dioulasso and Ouagadougou; Area 3: Ouagadougou, Boromo and Gourcy.

**Table 3:** Pooled prevalence and distribution of *Salmonella* serotypes.

*Salmonella* Serotypes Prevalence					Source-Specific Prevalence % (95% CI)			
Serotypes	No. of Study	Prevalence % (95% CI)	Q I^2^	I^2^	Human	Animal	Environment	Food
*S.* Derby	9	23.92 (20.76–27.39)	71.76	88.85	-	14.62 (11.54–18.36)	-	30.45 (19.80–43.70)
*S.* Croft II	1	18.75 (6.17–44.75)	0.00	0.00	-	-	18.75 (6.17–44.75)	-
*S.* Jodhpur II	1	18.75 (6.17–44.75)	0.00	0.00	-	-	18.75 (6.17–44.75)	-
*S.* Tennessee	3	16.35 (10.42–24.73)	46.21	95.67	-	0.79 (0.25–2.41)		27.74 (6.89–66.59)
*S.* Muenster	4	10.54 (7.99–13.80)	3.35	10.32	5.26 (1.32–18.76)	11.26 (8.45–14.84)	--	2.78 (0.39–17.26)
*S.* Typhimurium	5	10.42 (7.78–13.83)	39.29	89.82	21.27 (13.45–31.96)	2.09 (1.05–4.13)	-	12.03 (7.80–18.08)
*S.* Tilene	4	10.03 (4.94–19.28)	11.50	73.92	-	1.75 (0.25–11.43)	3.23 (0.45–19.64)	10.50 (0.99–57.81)
*S.* Jodhpur	2	9.77 (3.71–23.34)	0.26	0.00	-	-	12.50 (3.14–38.60)	7.69 (1.93–26.07)
*S.* Chester	4	9.07 (7.01–11.65)	2.39	0.00	-	8.38 (5.99–11.61)	3.23 (0.45–19.64)	10.78 (7.13–15.97)
*S.* Hato	7	8.31 (6.38–10.75)	6.78	11.50	-	7.63 (5.38–10.71)		9.12 (5.75–14.15)
*S.* Kentucky	7	7.69 (4.56–12.67)	14.27	57.97	6.67 (0.93–35.20)	1.75 (0.25–11.43)	18.75 (6.17–44.75)	4.53 (1.23–15.29)
*S.* Cubana	5	7.57 (3.81–14.48)	3.89	0.00	8.93 (2.89–24.47)	1.75 (0.25–11.43)	12.50 (3.14–38.60)	7.69 (1.93–26.07)
*S.* Virchow	4	7.03 (3.50–13.63)	14.40	79.17	13.33 (5.04–30.84)	0.26 (0.04–1.83)	9.68 (3.15–26.06)	-
*S.* Drac	2	6.86 (4.84–9.64)	0.25	0.00	-	6.86 (4.84–9.64)	-	-
*S.* Hvittingsfoss	2	6.74 (1.68–23.41)	0.51	0.00	6.74 (1.68–23.41)	-	-	-
*S.* Poona	7	6.67 (4.24–10.33)	26.11	77.02	15.83 (7.69–29.81)	1.31 (0.55–3.11)	16.13 (6.88–33.37)	3.27 (0.82–12.15)
*S.* Colindale	2	5.69 (2.34–13.22)	12.61	92.07	-	0.26 (0.04–1.83)	12.90 (4.93–29.74)	-
*S.* Essen	3	4.60 (1.90–10.68)	5.63	64.48	-	-	12.50 (3.14–38.60)	2.07 (0.34–11.61)
*S.* Bredeney	5	4.55 (2.87–7.12)	11.71	65.85	-	4.02 (2.33–6.83)	3.23 (0.45–19.64)	7.42 (2.81–18.17)
*S.* Duisburg	3	4.26 (1.37–12.48)	1.77	0.00	6.74 (1.68–23.41)	1.75 (0.25–11.43)	-	-
*S.* Nima	3	3.36 (1.74–6.41)	14.46	86.17	-	1.05 (0.39–2.76)	-	6.03 (0.73–35.88)
*S.* Brancaster	6	3.34 (2.08–5.32)	9.80	48.96	-	2.19 (0.98–4.83)	-	4.20 (2.34–7.42)
*S.* Agona	5	2.28 (1.19–4.34)	6.44	37.89	-	2.01 (0.90–4.42)	-	2.94 (0.95–8.73)
*S.* Ouakam	5	1.77 (0.73–4.21)	8.67	53.85	6.74 (1.68–23.41)	0.26 (0.04–1.83)	-	1.28 (0.32–4.96)
*S.* Eastbourne	4	1.57 (0.87–2.82)	1.11	0.00	-	1.56 (0.84–2.87)	-	1.75 (0.25–11.43)
*S.* Fresno	3	1.43 (0.64–3.15)	1.59	0.00	4.17 (0.58–24.35)	1.16 (0.48–2.76)	-	-
*S.* Korlebu	2	0.78 (0.25–2.40)	0.98	0.00	-	0.78 (0.25–2.40)	-	-

**Table 4:** Multidrug-resistant *Salmonella* serotypes from different sources *.

*Salmonella* Serotypes	Origine		
	Animals	Food	Humans
Brancaster			
Enteritidis			
Kentucky			
Typhimurium			
Hato			
Urbana			
Chester			
Derby			
Groupe Z			
Groupe B			
Virchow			
Poona			
Duisburg			
Ouakam			
*Salmonella* spp.			

Legend: The black box indicates multidrug-resistant *Salmonella* serotypes. * Data reported from the 28 references.

**Table 5:** Percentage of pooled resistance rates of antimicrobials to *Salmonella* isolates from humans, animals, food, and the environment.

Antibiotics	No. of Study	Prevalence % (95%)	Q	I^2^	Source-Specific Prevalence % (95% CI)			
					Animals	Food	Humans	Environment
Nalidixic acid	11	12.62 (9.88–16.00)	44.69	77.62	0.71 (0.21–2.44)	13.75 (10.36–18.02)	17.08 (10.15–27.32)	-
Amoxicillin	2	96.01 (87.16–98.84)	0.03	0	-	-	96.01 (87.16–98.84)	-
Amoxicillin and clavulanic acid	9	46.41 (41.08–51.82)	128.95	93.8	52.63 (31.11–73.22)	41.67 (35.97–47.60)	72.30 (58.81–82.68)	-
Ampicillin	6	15.26 (11.77–19.54)	59.2	91.55	1.83 (0.88–3.79)	15.17 (10.75–20.99)	41.92 (28.73–56.37)	-
Aztreonam	8	21.78 (17.35–26.97)	49.74	85.93	5.26 (0.74–29.39)	14.72 (10.84–19.68)	43.91 (32.38–56.13)	-
Cephalothin	1	54.65 (44.08–64.82)	0	0	-	54.65 (44.08–64.82)	-	-
Cefamandole	1	13.95 (8.10–22.98)	0	0	-	13.95 (8.10–22.98)	-	-
Cefepime	3	56.30 (43.86–68.00)	8.91	77.55	-	-	56.30 (43.86–68.00)	-
Cefixime	2	49.82 (37.52–62.14)	1.13	11.48	-	-	49.82 (37.52–62.14)	-
Cefotaxime	3	45.75 (35.20–56.70)	0.99	0	36.84 (18.68–59.70)	-	48.38 (36.27–60.69)	-
Ceftriaxone	7	21.26 (15.58–28.31)	70.85	91.53	5.26 (0.74–29.39)	3.80 (2.05–6.91)	48.57 (36.64–60.67)	-
Cephalexin	3	10.91 (7.29–16.03)	11.16	82.08	-	10.91 (7.29–16.03)	-	-
Chloramphenicol	11	15.56 (12.15–19.71)	134.85	92.58	1.63 (0.76–3.47)	3.23 (1.92–5.38)	43.78 (34.77–53.22)	-
Ciprofloxacin	8	11.85 (8.02–17.16)	17.21	59.32	5.26 (0.74–29.39)	9.43 (5.24–16.37)	15.77 (9.14–25.84)	-
Colistin sulfate	5	26.68 (21.40–32.72)	21.17	81.1	15.79 (5.18–39.15)	18.58 (13.59–24.88)	48.38 (36.27–60.69)	-
Erythromycin	2	98.34 (89.10–99.77)	0.17	0	97.50 (70.19–99.85)	98.89 (84.57–99.93)	-	-
Gentamicin	6	21.06 (16.93–25.88)	10.92	54.2	-	20.46 (15.88–25.95)	23.02 (14.73–34.10)	-
Imipenem	2	25.80 (14.68–41.27)	12.19	91.8	-	-	25.80 (14.68–41.27)	-
Kanamycin	1	5.56 (1.39–19.67)	0	0	-	5.56 (1.39–19.67)	-	-
Mecillinam	1	0.52 (0.13–2.07)	0	0	0.52 (0.13–2.07)	-	-	-
Netilmicin	1	1.89 (0.27–12.21)	0	0	-	-	1.89 (0.27–12.21)	-
Norfloxacin	2	20.89 (10.04–38.45)	3.81	73.78	-	7.69 (1.93–26.07)	33.33 (14.60–59.40)	-
Piperacillin	2	30.79 (20.56–43.33)	0.35	0	-	-	30.79 (20.56–43.33)	-
Piperacillin and Tazobactam	1	1.89 (0.27–12.21)	0	0	-	-	1.89 (0.27–12.21)	-
Spectinomycin	1	8.33 (2.71–22.86)	0	0	-	8.33 (2.71–22.86)	-	-
Streptomycin	8	11.64 (9.39–14.36)	14.46	51.58	9.51 (6.98–2.82)	12.39 (8.74–17.27)	33.33 (17.63–53.88)	6.45 (1.62–22.42)
Sulfonamides	4	10.01 (6.68–14.75)	36.35	91.75	1.83 (0.88–3.79)	16.37 (8.74–28.56)	33.33 (17.63–53.88)	-
Tetracycline	14	37.23 (32.53–42.18)	248.35	94.77	7.35 (4.35–2.14)	38.70 (32.98–44.74)	70.75 (59.36–80.02)	-
Ticarcillin	3	22.46 (12.72–36.53)	14.55	86.25	42.11 (22.63–64.39)	7.93 (2.94–19.67)	-	-
Tobramycin	2	33.87 (23.24–46.43)	0	0	-	-	33.87 (23.24–46.43)	-
Trimethoprim	2	7.56 (4.36–12.80)	33.25	96.99	1.57 (0.71–3.45)	-	33.33 (17.63–53.88)	-
Trimethoprim and Sulfamethoxazole	9	16.24 (12.36–21.05)	56.45	85.83	10.53 (2.65–33.74)	5.54 (3.50–8.65)	36.33 (26.77–47.12)	
MDR	10	29 (15–50)		94.34	-	-	-	-

## Data Availability

The data presented in this study are available upon request from the corresponding author.

## Supplementary Materials

The following supporting information can be downloaded at: https://www.mdpi.com/article/10.3390/antibiotics13060556/s1, Supplementary Material S1: Records list after duplicates removed; Supplementary Material S2: Serotypes and isolates.

## References

[1] WHO (1991). Urbanisation et Sante, Aperçu de la Situation Mondiale.

[2] Kone D.B., Kouamé F., Dongo K. (2008). Analyse de la situation de l’environnement sanitaire des quartiers défavorisés dans le tissu urbain de Yopougon a Abidjan, Côte d’Ivoire. VertigO.

[3] de Truchis P., de Truchis A. (2007). [Acute infectious diarrhea]. Presse Med..

[4] Nikiema M.E.M., Pardos de la Gandara M., Compaore K.A.M., Ky Ba A., Soro K.D., Nikiema P.A., Barro N. (2021). Contamination of street food with multidrug-resistant Salmonella, in Ouagadougou, Burkina Faso. PLoS ONE.

[5] Rotimi V.O., Jamal W., Pal T., Sonnevend A., Dimitrov T.S., Albert M.J. (2008). Emergence of multidrug-resistant *Salmonella* spp. and isolates with reduced susceptibility to ciprofloxacin in Kuwait and the United Arab Emirates. Diagn. Microbiol. Infect. Dis..

[6] Hennekinne J.-A., Herbin S., Firmesse O., Auvray F. (2015). European Food Poisoning Outbreaks Involving Meat and Meat-based Products. Procedia Food Sci..

[7] Majowicz S.E., Musto J., Scallan E., Angulo F.J., Kirk M., O’Brien S.J., Jones T.F., Fazil A. (2010). The global burden of nontyphoidal Salmonella gastroenteritis. Clin. Infect. Dis..

[8] Zeng X., Lv S., Qu C., Lan L., Tan D., Li X., Bai l. (2020). Serotypes, antibiotic resistance, and molecular characterization of non-typhoidal salmonella isolated from diarrheic patients in Guangxi Zhuang Autonomous Region, China, 2014–2017. Food Control.

[9] Koffi A.R., Ouassa T., Dadié A., Karou T.G., Koffi M.D. (2012). Sérotypes et profils d’antibio-résistance de Salmonella suspectées d’origine alimentaire et isolées chez des patients diarrhéiques à Abidjan, Côte d’Ivoire. Médecine d’Afrique Noire.

[10] Post A.S., Diallo S.N., Guiraud I., Lompo P., Tahita M.C., Maltha J., Van Puyvelde S., Mattheus W., Ley B., Thriemer K. (2019). Supporting evidence for a human reservoir of invasive non-Typhoidal Salmonella from household samples in Burkina Faso. PLoS Negl. Trop. Dis.

[11] Guibourdenche M., Roggentin P., Mikoleit M., Fields P.I., Bockemühl J., Grimont P.A., Weill F.X. (2010). Supplement 2003–2007 (No. 47) to the White-Kauffmann-Le Minor scheme. Res. Microbiol.

[12] Uche I.V., MacLennan C.A., Saul A. (2017). A Systematic Review of the Incidence, Risk Factors and Case Fatality Rates of Invasive Nontyphoidal Salmonella (iNTS) Disease in Africa (1966 to 2014). PLoS Negl. Trop. Dis.

[13] Chevalier P., van Driel M., Vermeire E. (2007). Hétérogénité dans les synthèses méthodiques et méta-analyses. Minerva.

[14] Hong S., Rovira A., Davies P., Ahlstrom C., Muellner P., Rendahl A., Olsen K., Bender J.B., Wells S., Perez A. (2016). Serotypes and Antimicrobial Resistance in *Salmonella enterica* Recovered from Clinical Samples from Cattle and Swine in Minnesota, 2006 to 2015. PLoS ONE.

[15] Kagambèga A., Hiott L.M., Boyle D.S., McMillan E.A., Sharma P., Gupta S.K., Ramadan H., Cho S., Humayoun S.B., Woodley T.A. (2021). Serotyping of sub-Saharan Africa Salmonella strains isolated from poultry feces using multiplex PCR and whole genome sequencing. BMC Microbiol..

[16] Afema J.A., Mather A.E., Sischo W.M. (2015). Antimicrobial Resistance Profiles and Diversity in Salmonella from Humans and Cattle, 2004–2011. Zoonoses Public Health.

[17] Odoch T., Wasteson Y., L’Abée-Lund T., Muwonge A., Kankya C., Nyakarahuka L., Tegule S. (2017). Prevalence, antimicrobial susceptibility and risk factors associated with non-typhoidal Salmonella on Ugandan layer hen farms. BMC Vet. Res..

[18] Mouttotou N., Ahmad D.S., Kamran Z., Koutoulis K. (2017). Prevalence, Risks and Antibiotic Resistance of Salmonella in Poultry Production Chain.

[19] Angelo K.M., Reynolds J., Karp B.E., Hoekstra R.M., Scheel C.M., Friedman C. (2016). Antimicrobial Resistance Among Nontyphoidal Salmonella Isolated from Blood in the United States, 2003–2013. J. Infect. Dis..

[20] Brands D.A., Inman A.E., Gerba C.P., Maré C.J., Billington S.J., Saif L.A., Levine J.F., Joens L.A. (2005). Prevalence of *Salmonella* spp. in oysters in the United States. Appl. Environ. Microbiol..

[21] Elkenany R.M., Eladl A.H., El-Shafei R.A. (2018). Genetic characterisation of class 1 integrons among multidrug-resistant *Salmonella* serotypes in broiler chicken farms. J. Glob. Antimicrob. Resist..

[22] Iwamoto M., Reynolds J., Karp B.E., Tate H., Fedorka-Cray P.J., Plumblee J.R., Hoekstra R.M., Whichard J.M. (2017). Ceftriaxone-Resistant Nontyphoidal Salmonella from Humans, Retail Meats, and Food Animals in the United States, 1996–2013. Foodborne Pathog. Dis..

[23] Dembélé R., Konaté A., Soulama I., Kagambèga A., Kaboré W., Cissé H., Traoré H., Traoré A.S., Barro N. (2018). Prevalence of Multidrug-resistant *Salmonella enterica* and associated factors among under five children with diarrhea in rural Burkina Faso. https://scientiaricerca.com/srcbmi/SRCBMI-03-00078.php.

[24] Konaté A. (2018). Prévalence, Facteurs Génétiques de Virulence et D’antibiorésistance de Escherichia coli et Salmonella spp. Isolés au Cours de Gastroenterites Infantiles au Burkina Faso.

[25] Moirongo R.M., Lorenz E., Ntinginya N.E., Dekker D., Fernandes J., Held J., Lamshöft M., Schaumburg F., Mangu C., Sudi L. (2020). Regional Variation of Extended-Spectrum Beta-Lactamase (ESBL)-Producing Enterobacterales, Fluoroquinolone-Resistant *Salmonella enterica* and Methicillin-Resistant *Staphylococcus aureus* Among Febrile Patients in Sub-Saharan Africa. Front. Microbiol..

[26] Somda N.S., Bonkoungou I., Samb-Ba B., Drabo M., Wane A., Sawadogo-Lingani H., Savadogo A. (2021). Diversity and antimicrobial drug resistance of non-typhoid *Salmonella* serotypes isolated in lettuce, irrigation water and clinical samples in Burkina Faso. J. Agric. Food Res..

[27] Bouda S., Kagambèga A., Bonifait L., Bako E., Cissé H., Ibrahim H.B., Le Gall F., Wereme-N’diaye A., Traore S.A., Chemaly M. (2019). Serotypes and Multiresistant *Salmonella* sp. from Chicken Eggs and Laying Hens in Burkina Faso. Int. J. Sci..

[28] Kagambèga A., Haukka K., Siitonen A., Traoré A.S., Barro N. (2011). Prevalence of *Salmonella enterica* and the hygienic indicator *Escherichia coli* in raw meat at markets in Ouagadougou, Burkina Faso. J. Food Prot..

[29] Kagambèga A., Barro N., Traoré A.S., Siitonen A., Haukka K. (2012). Characterization of *Salmonella enterica* and detection of the virulence genes specific to diarrheagenic Escherichia coli from poultry carcasses in Ouagadougou, Burkina Faso. Foodborne Pathog. Dis..

[30] Bawa I.H., Gertrude B.T., Serge B., Bouda S., Konaté A., Bako E., Kagambega A., Zongo C., Somda M., Savadogo A. (2016). Antimicrobial susceptibility of *Salmonella enterica* strains isolated from raw beef, mutton and intestines sold in Ouagadougou, Burkina Faso. J. Appl. Biosci..

[31] Gertrude B.T., Bawa I.H., Nzouankeu A., Bagré T.S., Traoré S.A., Barro N. (2015). Isolation, characterization and antibiotic susceptibility of *Escherichia coli* and *Salmonella* spp. isolated from local beverages (bissap, gnamakoudji) sold in Ouagadougou, Burkina Faso. Int. J. Biosci..

[32] Rouamba S.S., Somda N.S., Tapsoba F., Somda A., Ouédraogo M.-L., Elie K., Sangaré L., Savadogo A. (2022). Prevalence and antibioresistance of *Escherichia coli* and *Salmonella* isolated from lettuce and irrigation water in Ouagadougou, Burkina Faso. J. Life Sci. Biomed..

[33] Kagambèga A., Thibodeau A., Trinetta V., Soro D.K., Sama F.N., Bako É., Bouda C.S., Wereme N’Diaye A., Fravalo P., Barro N. (2018). *Salmonella* spp. and *Campylobacter* spp. in poultry feces and carcasses in Ouagadougou, Burkina Faso. Food Sci. Nutr..

[34] Gertrude B.T., Bawa H., Nzouankeu A., Serge B., Dembélé R., Bonkoungou I.J.O., Zongo C., Savadogo A., Traoré S.A., Barro N. (2014). Occurrence and antimicrobial susceptibility of Escherichia coli and Salmonella spp. Isolated from “zoom-koom” beverage and ice in Ouagadougou, Burkina Faso. Afr. J. Microbiol. Res..

[35] Dao J., Stenchly K., Traoré O., Amoah P., Buerkert A. (2018). Effects of Water Quality and Post-Harvest Handling on Microbiological Contamination of Lettuce at Urban and Peri-Urban Locations of Ouagadougou, Burkina Faso. Foods.

[36] Soubeiga A.P., Kpoda D.S., Compaoré M.K.A., Somda-Belemlougri A., Kaseko N., Rouamba S.S., Ouedraogo S., Traoré R., Karfo P., Nezien D. (2022). Molecular Characterization and the Antimicrobial Resistance Profile of *Salmonella* spp. Isolated from Ready-to-Eat Foods in Ouagadougou, Burkina Faso. Int. J. Microbiol..

[37] Bouda S., Kagambèga A., Bonifait L., Gall F., Bawa H., Bako E., Bagre T.S., Zongo C., Wereme-N’diaye A., Traore S.A. (2019). Prevalence and Antimicrobial Resistance of Salmonella enterica Isolated from Chicken and Guinea Fowl in Burkina Faso. Int. J. Microbiol. Biotechnol..

[38] Traoré O., Nyholm O., Siitonen A., Bonkoungou I.J., Traoré A.S., Barro N., Haukka K. (2015). Prevalence and diversity of Salmonella enterica in water, fish and lettuce in Ouagadougou, Burkina Faso. BMC Microbiol..

[39] Kagambèga A., Lienemann T., Aulu L., Traoré A.S., Barro N., Siitonen A., Haukka K. (2013). Prevalence and characterization of Salmonella enterica from the feces of cattle, poultry, swine and hedgehogs in Burkina Faso and their comparison to human Salmonella isolates. BMC Microbiol..

[40] Fousséni Y.B. (2011). Portage des Bactéries Entéropathogenes par des Insectes Domestiques Suceurs D’aliments cas de la Mouche Verte, de la Mouche Domestique et de la Blatte Germanique, Mémoire de DEA.

[41] Traoré O. (2009). Prévalence de Quelques Bactéries Entéropathogenes dans les Eaux de Fontaines, des Puits, Canaux et des Barrages Couramment Utilisées à Ouagadougou, Mémore de DEA.

[42] Bonkoungou I.J., Haukka K., Österblad M., Hakanen A.J., Traoré A.S., Barro N., Siitonen A. (2013). Bacterial and viral etiology of childhood diarrhea in Ouagadougou, Burkina Faso. BMC Pediatr..

[43] Dembélé R., Konaté A., Bonkoungou I., Kagambèga A., Konaté K., Serge B. (2014). Serotyping and antimicrobial susceptibility of Salmonella isolated from children under five years of age with diarrhea in rural, Burkina Faso. Afr. J. Microbiol. Res..

[44] Maltha J., Guiraud I., Kaboré B., Lompo P., Ley B., Bottieau E., Van Geet C., Tinto H., Jacobs J. (2014). Frequency of severe malaria and invasive bacterial infections among children admitted to a rural hospital in Burkina Faso. PLoS ONE.

[45] Kiemde F., Tahita M.C., Lompo P., Rouamba T., Some A.M., Tinto H., Mens P.F., Schallig H.D.F.H., van Hensbroek M.B. (2018). Treatable causes of fever among children under five years in a seasonal malaria transmission area in Burkina Faso. Infect Dis. Poverty.

[46] Sawadogo S., Diarra B., Bisseye C., Compaore T.R., Djigma F., Ouermi D., Ouattara A.S., Simporé S. (2017). Molecular Diagnosis of Shigella, Salmonella and Campylobacter by Multiplex Real-Time PCR in Stool Culture Samples in Ouagadougou (Burkina Faso). Sudan J. Med. Sci..

[47] Al-Emran H.M., Krumkamp R., Dekker D.M., Eibach D., Aaby P., Adu-Sarkodie Y., Ali M., Rubach M.P., Bjerregaard-Andersen M., Crump J.A. (2016). Validation and Identification of Invasive Salmonella Serotypes in Sub-Saharan Africa by Multiplex Polymerase Chain Reaction. Clin. Infect Dis..

[48] Dembélé R. (2010). Epidemiologie et Caractérisation Biochimique des Entéropathogènes Responsables de Diarrhée Chez Les Enfants de 0 à 5 ans en Milieu Rural au Burkina Faso, Mémoire deDEA.

[49] Alcaine S.D., Warnick L.D., Wiedmann M. (2007). Antimicrobial resistance in nontyphoidal Salmonella. J. Food Prot..

[50] Parry C.M., Threlfall E.J. (2008). Antimicrobial resistance in typhoidal and nontyphoidal salmonellae. Curr. Opin. Infect. Dis..

[51] Nacer S., Ftouhy F., Saâdia N., Lkhider M. (2022). *Salmonella* spp., Entre l’aspect zoonotique et l’antibiorésistance, quel enjeu pour le secteur de l’aviculture la filière avicole?. Rev. Marocaine Sci. Agron. Vétérinaires.

[52] Talukder H., Roky S.A., Debnath K., Sharma B., Ahmed J., Roy S. (2023). Prevalence and Antimicrobial Resistance Profile of Salmonella Isolated from Human, Animal and Environment Samples in South Asia, A 10-Year Meta-analysis. J. Epidemiol. Glob. Health.

[53] Parkunan T., Ashutosh M., Sukumar B., Chera J.S., Ramadas S., Chandrasekhar B., Kumar S.A., Sharma R., Kumar M.S., De S. (2019). Antibiotic resistance, A cross-sectional study on knowledge, attitude, and practices among veterinarians of Haryana state in India. Vet. World.

[54] Roess A.A., Winch P.J., Akhter A., Afroz D., Ali N.A., Shah R., Begum N., Seraji H.R., El Arifeen S., Darmstadt G.L. (2015). Household Animal and Human Medicine Use and Animal Husbandry Practices in Rural Bangladesh, Risk Factors for Emerging Zoonotic Disease and Antibiotic Resistance. Zoonoses Public Health.

[55] Winfield M.D., Groisman E.A. (2003). Role of nonhost environments in the lifestyles of *Salmonella* and *Escherichia coli*. Appl. Environ. Microbiol..

[56] Alio Sanda A., Samna Soumana O., Maârouhi I., Ali D., Bakasso Y. (2017). Prévalence Et Diversité De Salmonella En Afrique, Analyse Qualitative Et Quantitative. Eur. Sci. J..

[57] Abate D., Assefa N. (2021). Prevalence and antimicrobial resistance patterns of Salmonella isolates in human stools and animal origin foods in Ethiopia, A systematic review and meta-analysis. Int. J. Health Sci..

[58] Al-Rifai R.H., Chaabna K., Denagamage T., Alali W.Q. (2019). Prevalence of enteric non-typhoidal Salmonella in humans in the Middle East and North Africa, A systematic review and meta-analysis. Zoonoses Public Health.

[59] Abebe W., Earsido A., Taye S., Assefa M., Eyasu A., Godebo G. (2018). Prevalence and antibiotic susceptibility patterns of Shigella and Salmonella among children aged below five years with Diarrhoea attending Nigist Eleni Mohammed memorial hospital, South Ethiopia. BMC Pediatr..

[60] Ameya G., Tsalla T., Getu F., Getu E. (2018). Antimicrobial susceptibility pattern, and associated factors of Salmonella and Shigella infections among under five children in Arba Minch, South Ethiopia. Ann. Clin. Microbiol. Antimicrob..

[61] Wilson C.N., Pulford C.V., Akoko J., Perez Sepulveda B., Predeus A.V., Bevington J., Duncan P., Hall N., Wigley P., Feasey N. (2020). Salmonella identified in pigs in Kenya and Malawi reveals the potential for zoonotic transmission in emerging pork markets. PLoS Negl. Trop. Dis..

[62] Olufemi O., Oluwadaisi O., Atanda O., Atinuke D., Otesile E. (2019). Prevalence and antimicrobial resistance of Salmonella and Yersinia from wildlife. Rev. D’élevage Médecine Vétérinaire Pays Trop..

[63] Gutema F.D., Agga G.E., Abdi R.D., De Zutter L., Duchateau L., Gabriël S. (2019). Prevalence and Serotype Diversity of Salmonella in Apparently Healthy Cattle, Systematic Review and Meta-Analysis of Published Studies, 2000–2017. Front. Vet. Sci..

[64] Laufer A.S., Grass J., Holt K., Whichard J.M., Griffin P.M., Gould L.H. (2015). Outbreaks of Salmonella infections attributed to beef—United States, 1973–2011. Epidemiol. Infect..

[65] Elgroud R., Zerdoumi F., Benazzouz M., Bouzitouna C., Granier S., Brisabois A., Dufour B., Millemann Y. (2008). Contaminations du poulet de chair par les salmonelles non typhiques dans les élevages et abattoirs de la wilaya de Constantine. Sci. Technol. C Biotechnol..

[66] Sacko B., Sidibe S., Kouyate M., Traore O., Sow I., Coulibaly K., Babana A.H. (2019). Prevalence of *Salmonella* spp. Isolated from Poultry Products (Carcasses and Eggs) from Markets and Slaughterhouses of Bamako. Am. J. Biomed. Life Sci..

[67] Al-Rifai R.H., Chaabna K., Denagamage T., Alali W.Q. (2020). Prevalence of non-typhoidal Salmonella enterica in food products in the Middle East and North Africa, A systematic review and meta-analysis. Food Control.

[68] Nikiema P., Barro N., Razack B., Itsiembou Y., Savadogo A., Tidiane O., Philippe N.A. (2007). Street-Vended Foods Improvement, Contamination Mechanisms and Application of Food Safety Objective Strategy, Critical Review. Pak. J. Nutr..

[69] Avril J.L., Dabernat H., Denis F., Monteil H. (1992). Bactériologie Clinique.

[70] Denis F. (2007). Bactériologie Médicale, Techniques Usuelles.

[71] Agnandji P., Sanni A., Ayi-Fanou L. (2018). Analyse des pratiques phytosanitaires en maraîchage dans les zones intra-urbaines (Cotonou) et péri-urbaines (Sèmè-kpodji) au Sud-Bénin. Rev. Afr. d’Environnement d’Agriculture.

[72] Ndiaye M., Pfeifer H.-R., Niang S., Dieng Y., Tonolla M., Peduzzi R. (2010). Impacts de l’utilisation des eaux polluées en agriculture urbaine sur la qualité de la nappe de Dakar (Sénégal). VertigO.

[73] Alcaide E., Martinez J.P., Garay E. (1984). Comparative study on Salmonella isolation from sewage—Contaminated natural waters. J. Appl. Bacteriol..

[74] Abulreesh H. (2012). Salmonellae in the Environment.

[75] Liu H., Whitehouse C.A., Li B. (2018). Presence and Persistence of Salmonella in Water, The Impact on Microbial Quality of Water and Food Safety. Front. Public Health.

[76] Gestion Des Eaux Usées: Le Point sur Les Abattoirs du Québec. https://www.proquest.com/openview/f387f89b40415a1e6e312d3a66cd0a1a/1?pq-origsite=gscholar&cbl=237302.

[77] Angulo F.J., Nunnery J.A., Bair H.D. (2004). Antimicrobial resistance in zoonotic enteric pathogens. Rev. Sci. Tech..

[78] Fernandes S.A., Tavechio A.T., Ghilardi Â.C.R., Almeida E.A., Silva J., Camargo C.H., Tiba-Casas M.R. (2022). *Salmonella enterica* serotypes from human and nonhuman sources in Sao Paulo State, Brazil, 2004–2020. Rev. Inst. Med. Trop. Sao Paulo.

[79] Ramatla T., Tawana M., Onyiche T.E., Lekota K.E., Thekisoe O. (2022). One Health Perspective of Salmonella Serovars in South Africa Using Pooled Prevalence, Systematic Review and Meta-Analysis. Int. J. Microbiol..

[80] Evangelopoulou G., Kritas S., Govaris A., Burriel A.R. (2013). Animal salmonelloses, a brief review of? host adaptation and host specificity? of *Salmonella* spp.. Vet. World.

[81] Kahsay A.G., Dejene T.A., Kassaye E. (2023). A Systematic review on Prevalence, Serotypes and Antibiotic resistance of Salmonella in Ethiopia, 2010–2022. Infect. Drug Resist..

[82] Gao F., Huang Z., Xiong Z., Zheng H., Deng Q., Zhong H., Zhu S., Long Y., Wang J. (2023). Prevalence, serotype, and antimicrobial resistance profiles of children infected with Salmonella in Guangzhou, southern China, 2016–2021. Front. Pediatr..

[83] Oneko M., Kariuki S., Muturi-Kioi V., Otieno K., Otieno V.O., Williamson J.M., Folster J., Parsons M.B., Slutsker L. (2015). Emergence of Community-Acquired, Multidrug-Resistant Invasive Nontyphoidal Salmonella Disease in Rural Western Kenya, 2009–2013. Clin. Infect. Dis..

[84] Tadesse G., Tessema T.S. (2014). A meta-analysis of the prevalence of Salmonella in food animals in Ethiopia. BMC Microbiol..

[85] Nolan J.P., Monsieurs K.G., Bossaert L., Böttiger B.W., Greif R., Lott C., Madar J., Olasveengen T.M. (2020). European Resuscitation Council COVID-19 guidelines executive summary. Resuscitation.

[86] Sana B., Kaboré A., Hien H., Zoungrana B.E., Meda N. (2019). [Study considering the use of medicines in children receiving free care]. Pan. Afr. Med. J..

[87] Chereau F., Opatowski L., Tourdjman M., Vong S. (2017). Risk assessment for antibiotic resistance in South East Asia. BMJ.

[88] Ramatla T., Tawana M., Onyiche T.E., Lekota K.E., Thekisoe O. (2021). Prevalence of Antibiotic Resistance in Salmonella Serotypes Concurrently Isolated from the Environment, Animals, and Humans in South Africa, A Systematic Review and Meta-Analysis. Antibiotics.

[89] Antunes P., Machado J., Peixe L. (2006). Illegal use of nitrofurans in food animals, Contribution to human salmonellosis?. Clin. Microbiol. Infect..

[90] Manning J., Gole V., Chousalkar K. (2015). Screening for Salmonella in backyard chickens. Prev. Vet. Med..

[91] Abukhattab S., Taweel H., Awad A., Crump L., Vonaesch P., Zinsstag J., Hattendorf J., Abu-Rmeileh N.M.E. (2022). Systematic Review and Meta-Analysis of Integrated Studies on Salmonella and Campylobacter Prevalence, Serovar, and Phenotyping and Genetic of Antimicrobial Resistance in the Middle East-A One Health Perspective. Antibiotics.

[92] Eng S.-K., Pusparajah P., Ab Mutalib N.-S., Ser H.-L., Chan K.-G., Lee L.-H. (2015). *Salmonella*, a review on pathogenesis, epidemiology and antibiotic resistance. Front. Life Sci..

[93] El-Tayeb M.A., Ibrahim A.S.S., Al-Salamah A.A., Almaary K.S., Elbadawi Y.B. (2017). Prevalence, serotyping and antimicrobials resistance mechanism of *Salmonella enterica* isolated from clinical and environmental samples in Saudi Arabia. Braz. J. Microbiol..

[94] Vaez H., Ghanbari F., Sahebkar A., Khademi F. (2020). Antibiotic resistance profiles of Salmonella serotypes isolated from animals in Iran, a meta-analysis. Iran. J. Vet. Res..

[95] Djeghout B., Ammar A., Paglietti B., Langridge G., Rubino S. (2017). An Algerian perspective on non-typhoidal Salmonella infection. J. Infect. Dev. Ctries..

[96] Zhao X., Hu M., Zhang Q., Zhao C., Zhang Y., Li L., Qi J., Luo Y., Zhou D., Liu Y. (2020). Characterization of integrons and antimicrobial resistance in Salmonella from broilers in Shandong, China. Poult. Sci..

[97] Moher D., Shamseer L., Clarke M., Ghersi D., Liberati A., Petticrew M., Shekelle P., Stewart L.A. (2015). Preferred reporting items for systematic review and meta-analysis protocols (PRISMA-P) 2015 statement. Syst. Rev..

[98] Egger M., Davey Smith G., Schneider M., Minder C. (1997). Bias in meta-analysis detected by a simple, graphical test. BMJ.

