# Supplementary material for: Characteristics of Nontyphoid Salmonella Isolated from Human, Environmental, Animal, and Food Samples in Burkina Faso: A Systematic Review and Meta-Analysis
Source: Antibiotics (Basel). 2024 Jun 13;13(6):556. doi: 10.3390/antibiotics13060556 (PMC11200751; doi:10.3390/antibiotics13060556)
Supplement: Supplementary file 1 [file antibiotics-13-00556-s001.zip › Supplementary material S1.pdf]

Records list after duplicates removed

1. Abasse, O. G.; Cissé, H.; Boukaré, K.; Sampo, E.; Stéphane, K. D.; Odetokun, I.; Sawadogo, A.; Imael, B.; Nestor, H.; Savadogo, A. (2021). Original article Spread and antibiotic resistance profile of pathogens isolated from human and hospital wastewater in Ouagadougou. *Microbes infect.* **2021**
2. Al-Emran, H.M.; Krumkamp, R.; Dekker, D.M.; Eibach, D.; Aaby, P.; Adu-Sarkodie, Y.; Ali, M.; Rubach, M.P.; Bjerregaard-Andersen, M.; Crump, J.A.; et al. Validation and Identification of Invasive Salmonella Serotypes in Sub-Saharan Africa by Multiplex Polymerase Chain Reaction. *Clin Infect Dis.* **2016**, *62*
3. Bagre, T.S. *Evaluation de la contamination par des résidus d'antibiotiques, des pathovars de Escherichia coli et Salmonela enterica du lait et des produits laitiers consommés au Burkina Faso.pdf.* Université de Ouagadougou I., 2016.
4. Bagre, T.S.; Assèta, K.; Hadiza, I.B.; Gertrude, B.T.; Réné, D.; Cheikna, Z.; Aly, S.; Hebib, A.; Alfred, S.T.; Nicolas, B. (2014). Antibiotic susceptibility of Escherichia coli and Salmonella strains isolated from raw and curds milk consumed in Ouagadougou and Ziniar, Burkina Faso. *Afr. j. microbiol. Res*. **2014**, *8*, 1012–1016.
5. Barro, N.; Abdoul, R.B.; Aly, S.; Amadou T.O.C., Jules, I.A.; Alfred S.T. Hygienic status assessment of dish washing waters, utensils, hands and pieces of money from street food processing sites in Ouagadougou (Burkina Faso). *Afr. J. Biotechnol*. **2006**, *5*, 1107–1112.
6. Bsadjo-Tchamba, G.; Bawa, H.I.; Nzouankeu, A.; Bangre, T.S.; Dembélé, R.; Bonkoungou, I.J.O.; Zongo, C.; Aly, S.; Traoré, A.S.; Nicolas, B. Occurrence and antimicrobial susceptibility of Escherichia coli and Salmonella spp. isolated from "zoom- koom" beverage and ice in Ouagadougou, Burkina Faso. *Afr. j. agric. Res.* **2011**, *6*, 6348–6353.
7. Bsadjo Tchamba, G.; Touwendsida, S.B.; Traoré, A.S.; Barro, N. Isolation, characterization and antibiotic susceptibility of Escherichia coli and salmonella spp. isolated from local beverages («bissap», «gnamakoudji») sold in Ouagadougou, BURKINA FASO. *Int. j. biosci.* **2015**, *6*, 112–119.
8. Bawa, I.; Bsadjo Tchamba, G.; Bagre, T.; Bouda, S.; Konate, A.; Bako, E.; Kagambega, A.; Zongo, C.; Somda, M.; Savadogo, A.; et al. Antimicrobial susceptibility of Salmonella enterica strains isolated from raw beef, mutton and intestines sold in Ouagadougou, Burkina Faso. *J. appl. Biosci.* **2015**, *95*, 8966.
9. Bawa, H.I. Etude de la susceptibilité des microorganismes aux antibiotiques couramment utilisés en Afrique de l'Ouest : cas des souches bactériennes ioslés des produits pathologiques et environnementaux (viandes et eaux usées) du Niger et du Burkina Faso.pdf.
10. Bonkoungou, I.J.O.; Haukka, K.; Österblad, M.; Hakanen, A.J.; Traoré, A.S.; Barro, N.; Siitonen, A. Bacterial and viral etiology of childhood diarrhea in Ouagadougou, Burkina Faso. *BMC Pediatr.* **2013**, *13*, 2–7.
11. Bouda, S.C.; Kagambèga, A.; Bonifait, L.; Bako, E.; Cisse, H.; Ibrahim, H.B.; Le Gall, F.; Aïssata Wereme-N'diaye, A.; Traore, S.A.; Chemaly, M.; et al. Serotypes and Multiresistant Salmonella sp . from Chicken Eggs and Laying Hens in Burkina Faso. *Int. J. Sci.* **2019**
12. Bouda, S.C.; Kagambèga, A.; Bonifait, L.; Le Gall, F.; Ibrahim, H.B.; Bako, E.; Bagre, T.S.; Zongo, C.; Aïssata Wereme-N'diaye, A.; Traore, S.A.; et al. Prevalence and Antimicrobial Resistance of Salmonella enterica Isolated from Chicken and Guinea Fowl in Burkina Faso. *JMB.* **2019**, *4*, 64–71.
13. Compaoré, M.K.A.; Yougbaré, V.M.; Dembélé, R.; Nikièma, F.; Elie, K.; Barro, N. Retrospective study of the contamination of exported sesame by Salmonella species from 2007 to 2017 in Burkina Faso. *Afr. j. agric. Res.* **2020**, *16*, 1141–1147.
14. Dao, J.; Stenchly, K.; Traoré, O.; Amoah, P.; Buerkert, A. Effects of Water Quality and Post-Harvest Handling on Microbiological Contamination of Lettuce at Urban and Peri-Urban Locations of Ouagadougou, Burkina Faso. *Foods*. **2018**, *7*, 1–13.
15. Dao, J. Effects of irrigation water quality on soil properties and crops in urban gardens of Ouagadougou, Burkina Faso (Issue December). Faculty of Organic Agricultural Sciences at Universität Kassel, Germany, 2014.
16. Dembélé, R. Epidemiologie et caractérisation biochimique des entéropathogènes responsables de diarrhée chez les enfants de 0 à 5 ans en milieu rural au Burkina Faso.pdf. Université de Ouagadougou I, 2010.
17. Dembélé, R.; Konaté, A.; Soulama, I.;, Kagambèga, A.; Wendpoulomdé K.; Cissé, H.; Traoré, O.; Traoré, A.; Nicolas, B. Prevalence of Multidrug-resistant Salmonella enterica and associated factors among under five children with diarrhea in rural Burkina Faso. *CBMI.* **2018**, *3*, 566–576.
18. Dembélé, R.; Konaté, A.; Bonkoungou, I.; Kagambèga A.; Konaté, K.; Bagré, S.; Traoré, A.; Barro, N. Serotyping and antimicrobial susceptibility of Salmonella isolated from children under five years of age with diarrhea in rural Burkina Faso. *Afr. J. Microbiol.* **2014**, 8, 3157–3163.
19. Dembélé, R.; Konaté, A.; Traoré, O.; Kaboré, W.A.D.; Soulama, I.; Kagambèga, A.; Traoré, A.S.; Guessennd, N.K.; Aidara-Kane, A.; Gassama-Sow, A.; et al. Extended spectrum beta-lactamase and fluoroquinolone resistance genes among Escherichia coli and Salmonella isolates from children with diarrhea, Burkina Faso. *BMC Pediatr.* **2020.**
20. Douamba, S.; Nagalo, K.; Tamini, L.; Traoré, I.; Kam, M.; Kouéta, F.; Yé, D. Syndromes drépanocytaires majeurs et infections associées chez l 'enfant au Burkina Faso. *Pan Afr Med J.* **2017**, *26*.

21. Fousséni, Y.B. Portage des bactéries entomopathogènes par des insectes domestiques suceurs d'aliments : cas des mouches verte, de la mouche domestique et de la blatte germanique.pdf. Université de Ouagadougou I., 2011.
22. Ilboudo, A.J.; Savadogo, A.; Barro, N.; Ouedraogo, M.; Traore, A.S. Qualité hygiénique de la viande utilisée en restauration collective dans trois restaurants universitaires de Ouagadougou (Burkina Faso). *Rev. Microbiol Ind San et Environn.* **2009**.
23. Kagambèga, A.; Lienemann, T.; Aulu, L.; Traoré, A.S.; Barro, N.; Siitonen, A.; Haukka, K. Prevalence and characterization of Salmonella enterica from the feces of cattle, poultry, swine and hedgehogs in Burkina Faso and their comparison to human Salmonella isolates. *BMC Microbiol.* **2013**, *13*.
24. Kagambèga, A.; Barro, N.; Traoré, A.S.; Siitonen, A.; Haukka, K. Characterization of Salmonella enterica and detection of the virulence genes specific to diarrheagenic Escherichia coli from poultry carcasses in Ouagadougou, Burkina Faso. *Foodborne Pathog Dis.* **2012**, *9*, 589-93.
25. Kagambèga, A.; Thibodeau, A.;, Trinetta, V.; Soro, D.K.;, Sama, F.N.;, Bako, É.; Bouda, C.S.; Wereme N'Diaye, A.; Fravalo, P.; Barro, N. Salmonella spp. and Campylobacter spp. in poultry feces and carcasses in Ouagadougou, Burkina Faso. *Food Sci Nutr*. **2018**, *6*, 1601–1606.
26. Kagambèga, A.; McMillan, E.A.; Bouda, S.C.; Hiott, L.M.; Ramadan, H.; Soro, D.K.; Sharma, P.; Gupta, S.K.; Barro, N.; Jackson, C.R.; et al. Resistance Genes, Plasmids, Multilocus Sequence Typing (MLST), and Phenotypic Resistance of Non-Typhoidal Salmonella (NTS) Isolated from Slaughtered Chickens in Burkina Faso. *Antibiotics (Basel).* **2022**, *11*, 1–18.
27. Kagambèga, A.; Lienemann, T.; Frye, J.G.; Barro, N.; Haukka, K. Whole genome sequencing of multidrug-resistant Salmonella enterica serovar Typhimurium isolated from humans and poultry in Burkina Faso. *Trop Med Health*. **2018**.
28. Kagambèga, A.; Belem, S.; McMillan, E.A.; Hiott, L.M.; Ramadan, H.; Soro, K.; Sharma, P.; Gupta, S.K.; Barro, N.; Jackson, C.R.; et al. (2021). Genome analysis of Salmonella strains isolated from imported frozen fish in Burkina Faso. *Annals of Microbiology.* **2021**.
29. Kagambega, A., Haukka, K.; Siitonen, A.; Traore, A.S.; Barro, N. Prevalence of Salmonella enterica and the Hygienic Indicator Escherichia coli in Raw Meat at Markets in Ouagadougou, Burkina Faso. *J Food Prot*. **2011**, *74*, 1547–1551.
30. Kiemde, F.; Tahita, M.C.; Lompo, P.; Rouamba, T.; Some, A.M.; Tinto, H.; Mens, P.F.; Schallig, H.D.F.H.; van Hensbroek, M.B. Treatable causes of fever among children under five years in a seasonal malaria transmission area in Burkina Faso. *Infect Dis Poverty*. **2018**, *7*.
31. Konaté, A. Prévalence, facteurs génétiques de virulence et d'antibiorésistance de Escherichia coli et Salmonella spp. isolés au cours de gastroenterites infantiles au Burkina Faso.pdf. Université de Ouagadougou I., 2018.
32. Maltha, J.; Guiraud, I.; Kaboré, B.; Lompo, P.; Ley, B.; Bottieau, E.; Van Geet, C.; Tinto, H.; Jacobs, J. Frequency of severe malaria and invasive bacterial infections among children admitted to a rural hospital in Burkina Faso. *PLoS One*. **2014**, *9*.
33. Marks, F.; von Kalckreuth, V.; Aaby, P.; Adu-sarkodie, Y.; Ahmed, M.; Tayeb, E.; Ali, M.; Aseff, A.; Baker, S.; Biggs, H.M.; et al. Incidence of invasive salmonella disease in sub-Saharan Africa: a multicentre population-based surveillance study. *Lancet Glob Health.* **2017**, *5*.
34. Moirongo, R.M.; Lorenz, E.; Ntinginya, N.E.; Dekker, D.; Fernandes, J.; Held, J.; Lamshöft, M.; Schaumburg, F.; Mangu. C.; Sudi, L.; Regional variation of extended-Spectrum Beta-Lactamase (ESBL) -Producing Enterobacterales, Fluoroquinolone-Resistant Salmonella enterica and Methicillin-Resistant Staphylococcus aureus Among Febrile Patients in Sub-Saharan Africa. *Front Microbiol.* **2020**.
35. Nikiema, M.E.M.; Pardos de la Gandara, M.; Compaore, K.A.M.; Ky Ba, A.; Soro, K.D.; Nikiema, P.A.; Barro, N.; Sangare, L.; Weill, F.X. Contamination of street food with multidrugresistant Salmonella, in Ouagadougou, Burkina Faso. *PLoS One*. **2021**, *16*.
36. Park, S.E.; Pham, D.T.; Pak, G.D.; Panzner, U.; Maria Cruz Espinoza, L.; von Kalckreuth, V.; Im, J.; Mogeni, O.D.; Schütt-Gerowitt, H.; Crump, J.A.; et al. The genomic epidemiology of multi-drug resistant invasive non-typhoidal *Salmonella* in selected sub-Saharan African countries. *BMJ global health.* **2021**, *6.*
37. Rouamba, S.S.; Somda, N.S.; Tapsoba, F.; Somda, A.; Ouédraogo, M.-L.P.; Kabré, E.; Sangaré, L.; Savadogo, A. Prevalence and antibioresistance of Escherichia coli and Salmonella isolated from lettuce and irrigation water in Ouagadougou, Burkina Faso. *J. Life Sci.* **2022**, *12*, 1–7.
38. Sawadogo, S.; Diarra, B.; Bisseye, C.; Compaore, T.R.; Djigma, F.W.; Ouermi, D.; Ouattara, A.S.; Simporé, J. (2017). Molecular Diagnosis of Shigella , Salmonella and Campylobacter by Multiplex Real-Time PCR in Stool Culture Samples in Ouagadougou (Burkina Faso). *SJMS*. **2017**, *12*, 163–173.
39. Somda, N.S.; Juste, I.; Bonkoungou, O.; Sambe-ba, B.; Soungalo, M.; Aziz, A. (2021). Diversity and antimicrobial drug resistance of non-typhoid Salmonella serotypes isolated in lettuce, irrigation water and clinical samples in Burkina Faso. *J AGR FOOD RES.* **2021**, *5*.
40. Somda, N.S. Serotypage et antibioresistance des souches de Salmonella spp. et de shigella spp. isolées chez les enfants de moins de 5 ans en milieu urbain et rural au Burkina Faso.pdf. Université de Ouagadougou I., 2013.
41. Somda, M.K.; Samake, S.;, Kabore, D.; Nikiema, M.; Mogmenga, I.; Dabire, Y.; Ouattara, A.; Keita, I.; Mihin, H.B.; Akakpo, A.Y.; et al. Assessment of Heavy Metals and Microbial Pollution of Lettuce (Lactuca sativa) Cultivated in Two Sites (Paspanga and Tanghin) of Ouagadougou , Burkina Faso. *J. environ. prot. Sci.* **2019**, 454–471.

42. Soubeiga, A.P.; Kpoda, D.S.; Compaoré, M.K.A.; Somda-Belemlougri, A.; Kaseko, N.; Rouamba, S.S.; Ouedraogo, S.; Traoré, R.; Karfo, P.; Nezien, D.; et al. Molecular Characterization and the Antimicrobial Resistance Profile of Salmonella spp . Isolated from Ready-to-Eat Foods in Ouagadougou, Burkina Faso. *Int J Microbiol*. **2022**.
43. Traoré, O. Prévalence de quelques bactéries entéropathogenes dans les eaux de fontaines, des puits, canaux et des barrages couramment utilisées à Ouagadougou.pdf. Université de Ouagadougou I., 2009.
44. Traoré, O.; Nyholm, O.; Siitonen, A.; Bonkoungou, I.J.O.; Traoré, A.S.; Barro, N.; Haukka, K. Prevalence and diversity of Salmonella enterica in water, fish and lettuce in Ouagadougou, Burkina Faso. *BMC Microbiol*. **2015**, *15*.
45. Zongo B,H.C. Evaluation des risques microbiologiques liés à la production du porc au four vendu dans la ville de Ouagadougou.pdf. Université de Ouagadougou., 2015.
